# A Hybrid Brain-Computer Interface-Based Mail Client

**DOI:** 10.1155/2013/750934

**Published:** 2013-04-18

**Authors:** Tianyou Yu, Yuanqing Li, Jinyi Long, Feng Li

**Affiliations:** ^1^School of Automation Science and Engineering, South China University of Technology, Guangzhou 510640, China; ^2^School of Computer and Communication Engineering, Changsha University of Science and Technology, Changsha 410114, China

## Abstract

Brain-computer interface-based communication plays an important role in brain-computer interface (BCI) applications; electronic mail is one of the most common communication tools. In this study, we propose a hybrid BCI-based mail client that implements electronic mail communication by means of real-time classification of multimodal features extracted from scalp electroencephalography (EEG). With this BCI mail client, users can receive, read, write, and attach files to their mail. Using a BCI mouse that utilizes hybrid brain signals, that is, motor imagery and P300 potential, the user can select and activate the function keys and links on the mail client graphical user interface (GUI). An adaptive P300 speller is employed for text input. The system has been tested with 6 subjects, and the experimental results validate the efficacy of the proposed method.

## 1. Introduction

Millions of patients suffer from motor disabilities, including amyotrophic lateral sclerosis (ALS), brainstem stroke, cerebral palys, or spinal cord injuries, that are referred, as locked-in syndrome (LIS). The maintenance of communication ability is an essential factor for improving the quality of life for this group of people, but these patients are often severely or totally paralyzed and can produce few signals for communicating with other people. Brain-computer interfaces (BCIs) directly translate brain activities recorded on the scalp into control commands by bypassing the normal neuromuscular pathways, thus enabling users with motor disabilities to convey their thoughts and intentions to the external world [1, 2]. Significant progress in this field has been achieved in recent decades, particularly in noninvasive BCIs (see [3, 4] for a comprehensive survey). Diverse types of real-world applications of BCIs have been developed, such as word spelling [5, 6], environment control [7], neural prothesis control [8], wheelchair control [9, 10], and gaming [11].

An important application for BCIs is communication [4], and electronic mail is a common and efficient communication tool in daily life. In addition to its use for normal communication purposes, an email account is commonly used as an ID on the Internet and in the real world. Thus, a BCI mail client is an application for LIS patients that promises to greatly extend their communication range. However, to our knowledge, no BCI-based mail client has yet been reported in the literature, perhaps because current mail clients are typically based on a computer mouse. Users operate these mail clients by performing two-dimensional movement control and clicking with the mouse. Of course, the implementation of such a BCI-based mouse is a tricky problem.

Hybrid BCIs, which use more than one brain signal or one brain signal and a different type of input to detect users' intentions, have attracted much attention in recent years [12]. Studies have shown that hybrid BCIs may yield a better performance than BCIs that use only one type of brain signal [13, 14]. In our previous studies [15, 16], we presented a hybrid BCI incorporating motor imagery-based ERD/ERS and the P300 potential for continuous 2D cursor movement control and target selection/clicking. This hybrid approach was successfully applied to a BCI browser for Internet surfing [17].

In this paper, we propose a BCI mail client as a novel application of the hybrid BCI. The control of this mail client is based on a BCI mouse consisting of two-dimensional cursor movement, target selection, and an adaptive P300 speller. Users are able to receive, read, write, reply to, and forward mails; they can attach files by moving the BCI mouse to the buttons or links on the mail client GUI and activating them. In particular, the horizontal movement of the mouse is controlled by mu/beta rhythm, the vertical movement of the mouse is controlled by P300 potential, and the target selection or click is controlled by a combination of the detection of P300 waves presented on one of the stop buttons and/or an idle state of mu/beta rhythm when the mouse is on a target (e.g., a menu, link, button, or text input). Furthermore, with an adaptive P300 speller, users can input the text content of a piece of mail.

This paper is organized as follows. The system paradigm, mouse control methodology and adaptive P300 detection are discussed in Section 2. The experimental paradigms and results are presented in Section 3. Further discussions on the system are found in Section 4. Finally, Section 5 concludes.

## 2. Methodology

**Figure 1:**
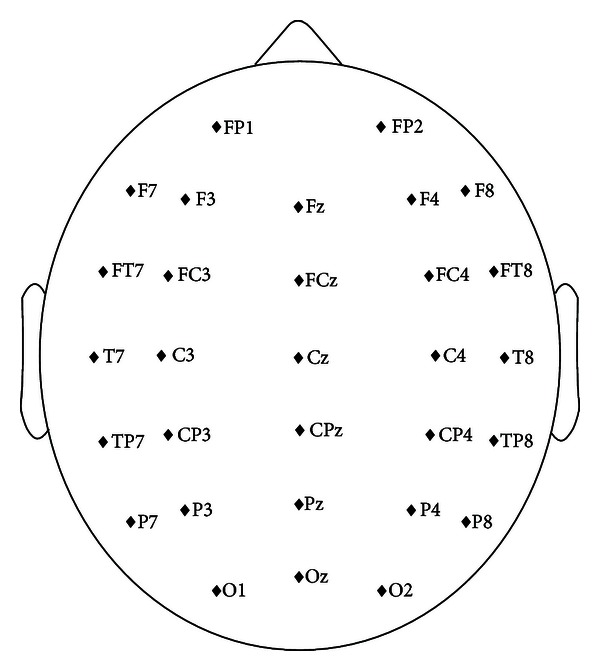
Names and distribution of electrodes.

Scalp EEG signals are recorded with a SynAmps2 amplifier using the 32-channel Quik-Cap (Neuroscan Compumedics, USA) at a sampling rate of 250 Hz and band-pass filtered between 0.05 and 40 Hz. Two channels, “HEOG” and “VEOG,” representing eye movements are excluded for signal processing. The remaining 30 channels are used without further channel selection and are shown in Figure 1 [15].

**Figure 2:**
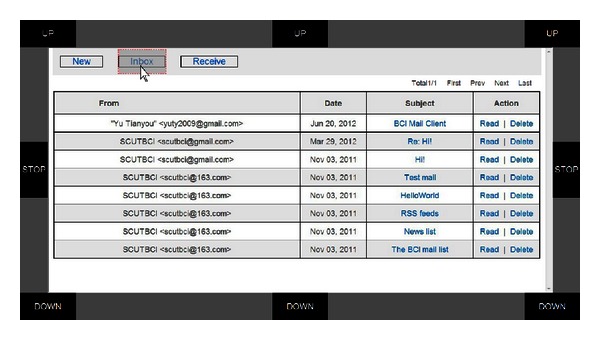
GUI of the BCI mail client in which the mail client is embedded in the center area with eight flashing buttons (“UP”, “DOWN,” and “STOP”) placed around it.

The graphical user interface (GUI) is presented in Figure 2. There are eight buttons on the margins of the GUI with 3 buttons labeled “UP” at the top, 3 buttons labeled “DOWN” at the bottom, and two buttons labeled “STOP” in the middle. During mouse control, these buttons flash in a random order to elicit P300 potentials when users focus their attention on one of them. The mail client is embedded in the center area.

The user controls the vertical movement of the mouse; that is, he/she moves the mouse up or down, by paying attention to one of the three “UP”/“DOWN” buttons. Meanwhile, the horizontal movement of the mouse is controlled by the user's motor imagery. Specifically, the user moves the mouse toward the right by imaging the movement of his/her right hand and vice versa for the left [15]. Once the cursor hits a target, it stops at the target for 2 seconds. During this period, the user makes a selection/rejection decision. The target selection or rejection is implemented using a hybrid feature composed of the motor imagery and P300 features. If the target is an intended one, the user can make a selection by paying attention to the “STOP” button on the left side without motor imagery (i.e., in a so-called idle state of motor imagery) for 2 seconds. Otherwise, if the target is of no interest, he/she can reject it by continuing motor imagery without paying attention to the “STOP” button (i.e., in a so-called idle state of P300). A detailed description appears in [16].

In the mail client GUI, each selectable target (including menu, link, button, and text input) is indicated by a translucent box that is placed on top of the target and becomes visible only when the mouse reaches it (see Figure 2). When a menu, link, or button is selected, the system executes corresponding command or follows the link, whereas when a text input is selected, the system switches to the P300 speller interface. Basic mail communication functions, such as receiving, reading, creating, replying, forwarding, and attaching files to mails, are included in this mail client. We describe these mail functions in the following list.

**Figure 3:**
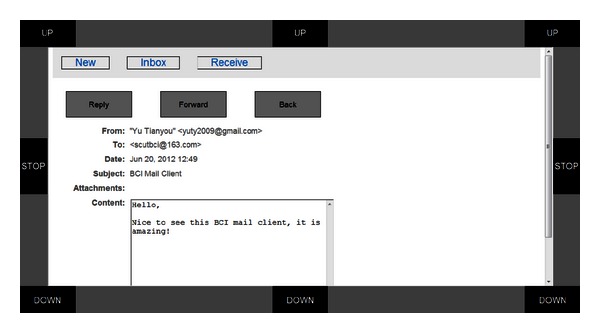
A piece of mail opened by the user. The user can read, reply, and/or forward this mail or return to the previous page.

**Figure 4:**
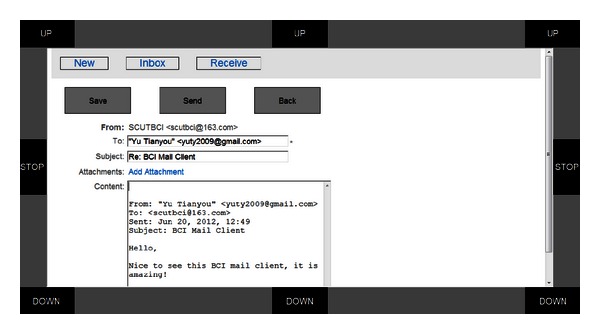
The replying interface of the mail client where users can input mail content and then save it as a draft or send it.

**Figure 5:**
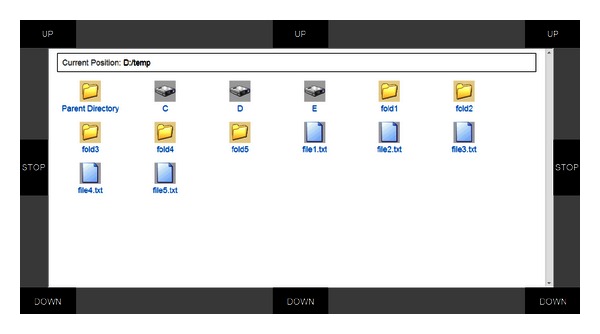
The file explorer interface for attaching files. With this interface, users can find the files that to be attached from the local computer.

1. *Receiving*: users move the mouse to the “Receive” button and select it. New incoming mails are then received (see Figure 2).
2. *Reading*, *replying and forwarding*: mails are listed in the “Inbox” with the titles linked to the mail contents (see Figure 2). By moving the mouse to a link and activating it, users open the corresponding mail content and read it. Users select the “Reply”/“Forward” button to reply or forward this mail (see Figure 3).
3. *Creating*: users move the mouse to select the “New” button in the GUI to initiate a piece of mail (see Figure 4).
4. *Writing*: after creating/replying/forwarding a piece of mail, users enter an interface similar to that shown in Figure 4. They fill or change the address in the “TO” box and the mail subject in the “Subject” box and input content into the “Content” box. They send this mail by selecting the “Send” button.
5. *File attaching*: when writing mail, users can select the “Add Attachment” link shown in Figure 4 to open a file explorer interface (see Figure 5). Users may traverse the local file system and select a file to attach.

### 2.1. Mouse Movement Control

The vertical and horizontal movements of the mouse are controlled by P300 and motor imagery, respectively. We briefly describe this method here, and additional details can be found in [15].

The position of the mouse is updated every 200 ms in our system. For vertical movement control of the mouse, the velocity is fixed and the direction *c*(*k*) of the *k*th update is determined by P300 detection. That is, if P300 potential is detected at one of the three “UP” buttons, *c*(*k*) is set to 1 and the mouse moves upward. If P300 potential is detected at one of the three “DOWN” buttons, *c*(*k*) is set to −1, and the mouse moves down. If P300 potential is detected at one of the two “STOP” buttons, *c*(*k*) is set to 0, and the mouse has no vertical movement. If no P300 potential is detected at any button, *c*(*k*) keeps its value, and the direction of vertical movement of the mouse does not change. For details of the P300 detection algorithm, readers can refer to [15]. Thus, the vertical coordinate of the mouse is updated according to the following equation:

(1) $$\begin{array}{c} y\left(k\right)=y\left(k-1\right)+c\left(k\right)v_{0},\;c\left(k\right)\in \left\{-1,0,1\right\}, \end{array}$$

where *v*_0_ is a speed constant which was set to 10 pixels in our experiments and can be tuned further according to users' performance.

For horizontal movement control, the position of the mouse in the *k*th update is determined by the classification result of left and right hands' motor imagery. Given the SVM classification score *f*(*k*), the horizontal coordinate of the mouse is updated according to

(2) $$\begin{array}{c} x\left(k\right)=x\left(k-1\right)+a_{x}\left[f\left(k-2\right)+f\left(k-1\right)+f\left(k\right)\right]+b_{x}, \end{array}$$

where the parameters *a*_*x*_ and *b*_*x*_ are calibrated immediately before an online experiment such that the absolute value of the difference (*x*(*k*) − *x*(*k* − 1)) is near zero when the subject is in an idle state of motor imagery (see [15]).

Users can move the mouse from an arbitrary initial position to an arbitrary target position with the above control method based on both P300 potential and mu/beta rhythm.

### 2.2. Target Selection or Rejection

A hybrid approach that combines both P300 potential and mu/beta rhythm for target selection, as described in [16], is employed in our system. Specifically, when the mouse reaches a target, the user can select it by focusing on the left flashing button “STOP” in the mail client GUI and making no motor imagery. Conversely, the user can reject it by continuing motor imagery and paying no attention to the “STOP” button. In other words, the detection of a P300 potential at the left “STOP” button with no motor imagery implies a selection of the currently reached target, whereas the detection of left/right motor imagery with no P300 potential at the same button indicates a rejection of this target. Given a segment of EEG signals, we extract both the P300 feature and the mu/beta rhythm feature then concatenate them to construct a hybrid feature vector that is classified by a trained SVM classifier. If the predicted label of this feature vector is 1, then the target is selected. Otherwise, the target is rejected.

### 2.3. Adaptive P300 Word Spelling

**Figure 6:**
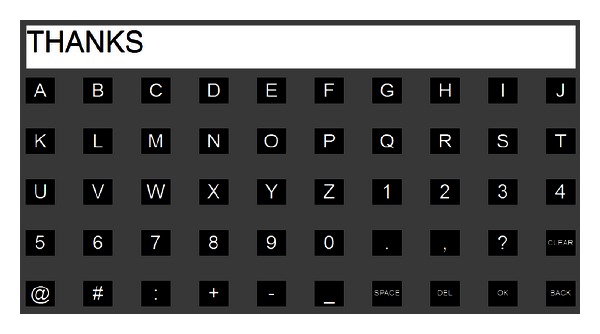
The GUI of the P300-based speller that is used for text input. Fifty buttons arranged in a 5 by 10 stimuli matrix corresponding to 45 characters and 5 function keys. “CLEAR” is used to clear all of the input characters, “SPACE” is used to input a space, “DEL” is used to delete the last input character, “OK” is used to return to the mail client interface with the input text, and “BACK” is used to cancel the input text and return the user to the mail client interface.

An adaptive P300 speller is integrated into the mail client for text input (see Figure 6). The method used for detecting P300 event-related potential is similar to that used in the cursor's vertical movement control but with different parameters. Fifty frequently used characters are included and placed in a 5 by 10 matrix. In contrast to the famous Farwell-Donchin's row column (RC) paradigm (see [5]), a single-character (SD) paradigm is employed; that is, characters are intensified one by one (see [18]). The interstimulus interval is 30 ms, and each character is intensified for 100 ms. This SD paradigm efficiently avoids the deterioration of P300 waves caused by the intensification of the intended row and column that is followed in the RC paradigm.

The P300 speller adaptively selects the number of epochs to average, according to the subject's current performance. Specifically, for all 30 channels, the 0–600 ms data segments after the stimulus onset were filtered in the range of 0.1–20 Hz and downsampled by taking the first time point from each piece of 6 consecutive ones. The obtained 30 data vectors (each for one channel) were then concatenated to construct a feature vector. A P300 stimulus round consists of 50 flashes, one for each character, which means 50 feature vectors. Give a new round, *l* + 1  (*l* is initialized to 0) rounds of feature vectors are classified by a Bayesian linear discriminant analysis (BLDA) classifier [19] that is trained previously to obtain identical rounds of classification scores. These scores are averaged by character to result in 50 scores, which are then normalized between 0 and 1. The final output *c*(*k*) of the *k*th round is determined by the difference of the maximum and second maximum score Δ*θ*, given a threshold *θ*_0_ and two parameters that limit the minimum (*l*_min⁡_) and maximum (*l*_max⁡_) numbers of rounds to average, respectively. In other words, when at least *l*_min⁡_ rounds of data are collected, and if Δ*θ* exceeds the threshold *θ*_0_ or *l* reaches *l*_max⁡_, the system outputs the character corresponding to the maximum score and *l* is reset to 0. Otherwise, the system continues to collect another round of data to determine the output.

According to the offline analysis, we have found that if *l*_min⁡_ < 3, the Δ*θ* is unlikely to exceed *θ*_0_ and the detection accuracy is very low. On the other hand, when *l*_max⁡_ > 8, the detection time is long and the newly incoming data usually does not change the detection result. Thus, to ensure both the spelling accuracy and speed, we set *l*_min⁡_ = 3 and *l*_max⁡_ = 8, respectively, in this paper. Meanwhile, the value of threshold *θ*_0_ is calculated from the P300 calibration data (see Section 3 for the details of how the calibration data is collected). Specifically, since the classification scores have been normalized between 0 and 1, we select *θ*_0_ from the range [0 1] with a step 0.05. Detection accuracy and information transfer rate (ITR) [2] are calculated, by 10-fold cross-validation, for different *θ*_0_ in the set {0,0.05,0.10,…, 1.0}. The formulation of ITR is as follows:

(3) B[bits/min⁡]  =M{log⁡2N+Plog⁡2P+(1−P)log⁡2[(1−P)(N−1)]},

where *N* is the number of characters (50 here), *P* denotes the detection accuracy, and *M* represents the number of decisions that are made per minute. Then, the value of *θ*_0_, which provides the highest ITR with an accuracy above 90% of the highest accuracy, is chosen as the *θ*_0_ that is used in the online spelling. Accordingly, *θ*_0_ is set between 0.2 and 0.3 in this paper.

## 3. Experiments and Results

Six subjects, aged from 23 to 30, participated in the online experiment. Two datasets were collected from each subject earlier to set the parameters of the three models as below. For Dataset I, each subject attended a P300 calibration session of 20 trials with the GUI of the P300 speller (see Figure 6). Specifically, in each trial, all 50 buttons flashed randomly and each button flashed 10 times. The subject was instructed to focus his attention on a given button. For Dataset II, in this calibration session, there were 3 classes of trials, corresponding to 3 separate tasks, performed by each subject. Each class contained 30 trials and each trial lasted for 4 seconds. In each trial, a cue (left/right/upward arrow) appeared to instruct a task, and the 8 buttons on the GUI shown in Figure 2 flashed randomly to evoke the P300 potential. The P300 stimuli were synchronized with the appearance of the cue; that is, the stimuli began when the arrow appeared and ended when it disappeared. When a left/right arrow appeared, the subject imagined left/right hand motor without paying attention to the flashing button, and the subject focused on the “STOP” button without any motor imagery when an upward arrow appeared. We used the above two datasets to establish the three models. In the first, the P300 model, Dataset I was used to construct a P300 classification model for text input and control of mouse's vertical movement. In the second, motor imagery model, the left/right motor imagery trials in Dataset II were used to set up the model for motor imagery detection. In the third, hybrid model, all of the trials in Dataset II were labeled as trials of selection (trials with an up arrow cue) or rejection (trials with left/right arrow), and these trials were further employed to train the model for target selection.

### 3.1. Online Experiment

In the online experiment, each of the six subjects was requested to receive mails and reply to the latest mail with an attachment, using the hybrid BCI-mail client to complete all these tasks. Specifically, the task for each subject contained the following 9 sequential operations.

**Figure 7:**
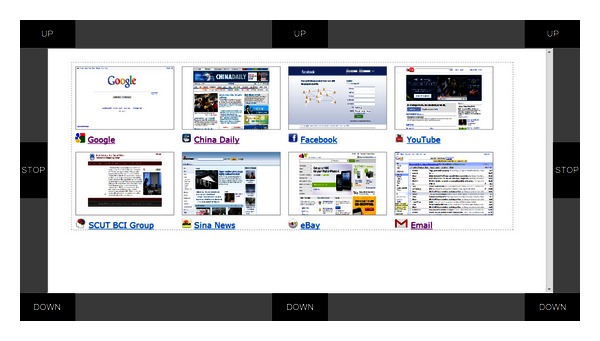
Initial home page containing the mail site and several other popular sites.

1. The subject first activated the mail client from the predefined home page (see Figure 7) using the BCI mouse.
2. After the mail client was opened, the subject moved the mouse toward the “Receive” button and selected it.
3. Once the receiving procedure finished, the mail client returned to the “Inbox.” The subject moved the mouse and opened the latest piece of mail.
4. The subject replied to the writer by moving the mouse to and selecting the “Reply” button after reading the mail.
5. The subject selected the “Add attachment” button to attach a file.
6. In the file list view shown in Figure 5, the subject selected the last file as an attachment.
7. The subject selected the “Content” box for text input. The system switched to the P300 speller interface (see Figure 6).
8. The subject input mail content, for example, a simple sentence, with the P300 speller.
9. After inputting text, the subject selected the “Back” button of the GUI of the speller to return to the previous interface. The subject moved the mouse to and selected the “Send” button to send this mail.

**Figure 8:**
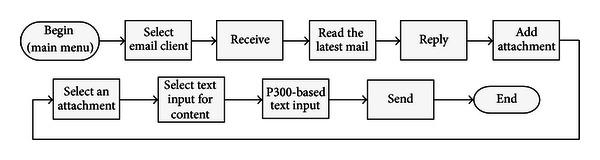
The online experiment flow including 9 operations.

Once these nine operations were completed, the mail client returned to the main menu for the next trial. The entire procedure is illustrated in Figure 8. Each subject repeated this procedure 5 times, which corresponded to 5 trials. For each trial, the subjects performed at least eight selections using the BCI mouse. If an unintended target was selected, the subject had to select the “Back” button or the menus in the main menu bar to return to the previous step. This procedure implied that more than eight selections might be required for each trial. During text input, the subject could select the function key “DEL” or “CLEAR” to delete typos.  Table 1 shows the results of the online experiment, including the number of trials for each subject, the average number of selection operations, the average number of input characters, the average time of text spelling, and the average time necessary for a complete trial.

### 3.2. Workload Evaluation

To acquire a more complete evaluation of this BCI mail client, subjective workload and satisfaction were assessed with NASA Task Load Index (NASA-TLX) questionnaires [20] following [21, 22]. The NASA-TLX contains six factors (shown in Table 2), each of which has 20 levels and is scored from 0 to 100 (see [20] for details of the NASA-TLX). Small score represents low workload and vice versa. The questionnaires were self-completed by these subjects independently after the online experiment. The scores are reported in Table 2. It follows from Table 2 that the average score for the factor “Temporal demand” is equal to 42.5 and that the average scores for the other factors are less than 40. This observation indicates that the BCI mail client is acceptable for all subjects. However, large variances appear for the scores in Table 2 because the subjects had different states and control strategies when using the mail client. However, the high scores for the factor “Temporal demand” given by most of the subjects indicates that we must further improve the speed of the system. The low scores for “Frustration” may indicate that these healthy subjects are interested in using this BCI mail client and that the results meet their expectations.

## 4. Data Analysis and Discussions

In the online experiment, the task including multiple mouse operations and text input was quite complicated. Using this comprehensive task, we assessed the BCI mail client. Our results show that most of the functions for mail communication were available in the BCI mail client. Most of the subjects could finish a trial within 450 seconds with only a small number of mistakes, and all of the subjects were able to complete this complex task within an acceptable period of time. The workload evaluation further showed that this BCI mail client was acceptable to the subjects.

For comparison, all of the subjects performed the identical task using their hands as usual. The average time across all of the subjects was 57 seconds for a trial. It took these subjects a relatively long time to complete the trial using our hybrid BCI mail client for three reasons. First, extra selection operations were required to correct the selection of an unintended target. Second, text inputting was time consuming. Third, switching between mouse operation mode and text-spelling mode, which was also performed through target selection, costed additional time.

To confirm that both the P300 and motor imagery were functioning properly in our system, we further analyzed these EEG features as we have in our previous studies [15–17]. By analyzing the P300 calibration data, significant P300 waves could be observed from related electrodes for all subjects (e.g., “CPz” for subject S1 and “Oz” for subject S2). Additionally, we can see from the topographies of CSP filters and patterns, which were trained on the left and right motor imagery trials of Dataset II, that channels in primary sensorimotor areas (e.g., “C3” and “C4”) were associated with the left and right hands' motor imageries in the experiment. However, in the hybrid condition, both P300 wave and event-related desynchronization (ERD) were observed in the corresponding electrodes. For detailed analysis methods and feature visualizations, see [15–17].

Note that simultaneously executing the dual tasks of the P300 and motor imagery may deteriorate the performance of P300 or motor imagery for many subjects because both tasks require attention. The solution to this problem was twofold in our study. First, we set the parameters *a*_*x*_ and *b*_*x*_ in model (2) as in Section 2 making the horizontal displacement of the cursor small when the subject was focusing on a flashing button without motor imagery. Second, we set a threshold *θ* in our P300 detection algorithm, as in [15]. The principle for setting the threshold was that the output of the P300 detection algorithm could exceed the threshold when the subject was paying attention to a flashing button; however, the threshold was difficult to exceed when the subject did not pay attention to a flashing button. If the output of the P300 detection algorithm did not exceed this threshold, the direction of the vertical movement of the cursor did not change. The two principles for setting parameters led to the following control strategy, particularly for those subjects who have difficulty performing the P300 task and motor imagery task simultaneously. The strategy is to choose to focus on the P300 task or motor imagery task to control the vertical or horizontal movement of the cursor separately. For instance, subjects can pay attention primarily to a flashing button to elicit P300 at the beginning of a trial or when they want to change the direction of the vertical movement of the cursor. In this case, the cursor is stable in the horizontal dimension. Once the vertical movement direction of the cursor is correct, the subjects pay attention primarily to motor imagery to control the horizontal movement, and the vertical movement direction of the cursor generally does not change because of the threshold for P300 detection. Such parameter-setting methods enable P300 and motor imagery to work separately or simultaneously, depending on the control strategy of the subject.

Although our experimental results show that this BCI mail client is capable of providing basic email communication, our system needs to be improved and extended in the future. First, the mail client itself can be extended to include several other functions, such as accessibility to a contact list and the outbox. Second, the algorithms for detecting P300 potential and motor imageries can be further optimized to reduce the execution time for each task. Third, in addition to the P300 potential and motor imagery, other modalities, such as SSVEP, may be incorporated into our system for more functions and better performance. Finally, it will be of great interest to verify whether this system works for paralyzed subjects.

## 5. Conclusions

This study presents a hybrid BCI-based mail client as a real-world application of BCIs. Common functions of a mail client, including receiving, reading, writing mails, and attaching files, are implemented in this system. The mail client is based on a hybrid BCI mouse. The BCI mouse control, including 2D movement control and target selection, is implemented using the P300 potential and motor imagery-related mu/beta rhythm. The user operates the mail client using the BCI mouse to select function keys. Furthermore, an adaptive P300 speller is incorporated for text input. Experimental results show that users have access to basic mail communication through this BCI mail client.

## Figures and Tables

**Table 1:** Results of online experiment. Each trial of this experiment includes 9 operations as shown in Figure 8. “No. selections” is the average number of selections performed in a trial, “No. characters” is the average number of actual input characters for text input, “Spelling time” is the average time for text input, and “Trial time” is the average time for a complete trial.

Subject	No. trials	No. selections	No. characters	Spelling time (s)	Trial time (s)
S1	5	8.0	8.2	101.33	414.62
S2	5	8.8	7.2	99.68	444.14
S3	5	8.25	8.0	96.88	422.10
S4	5	9.0	7.4	82.05	448.20
S5	5	8.8	7.8	94.41	444.28
S6	5	8.0	7.8	102.52	430.87

Average	5	8.48	7.73	96.15	434.04

**Table 2:** Scores of the NASA-TLX rating scales given by the subjects after using the BCI mail client.

Subjects	Mental demand	Physical demand	Temporal demand	Performance	Effort	Frustration
S1	25	10	40	25	30	10
S2	30	10	60	30	40	30
S3	10	0	50	25	35	20
S4	10	5	10	10	10	15
S5	60	10	60	75	75	10
S6	30	15	35	20	35	25

Average	27.5	8.3	42.5	30.8	37.5	18.3

STD	18.4	7.3	18.9	22.7	21.2	8.2
